# Frontiers in aging special issue: DNA repair and interventions in aging perspective on “loss of epigenetic information as a cause of mammalian aging”

**DOI:** 10.3389/fragi.2023.1199596

**Published:** 2023-07-05

**Authors:** Ethan D. Schaffer, Isabel Beerman, Rafael de Cabo, Robert M. Brosh

**Affiliations:** Translational Gerontology Branch, National Institute on Aging, National Institutes of Health, Baltimore, MD, United States

**Keywords:** aging, epigenetic, healthspan, mouse, genetic, double-strand break, DNA damage, gene expression

## Abstract

The recently published article in *Cell* by the Sinclair lab and collaborators entitled “Loss of Epigenetic Information as a Cause of Mammalian Aging” [1] implicates heritable changes in gene expression as the basis for aging, a postulate consistent with the emerging information theory of aging. Sinclair’s group and colleagues induced epigenetic changes, i.e., DNA and histone modifications, via double-strand breaks (DSBs) catalyzed by the I-Pol endonuclease at specific genomic loci. The genomic DNA breaks, introduced without inducing insertion or deletion mutations (indels) in a mouse model, were targeted to 19 non-coding regions and one region in ribosomal DNA (rDNA), the latter shown to not have a significant effect on the function or transcription of rDNA [1]. With that experimental model in place, the authors present experimental evidence supporting a model that epigenetic changes drive aging via this inducible DNA break mechanism. After demonstrating the phenotypic alterations of this accelerated aging, they attempt to reverse selective phenotypes by resetting the altered epigenetic landscape. Establishing a causal relationship between epigenetic changes and aging, and how this connection might be manipulated to overturn cellular features of aging, is provocative and merits further study.

## Epigenetics and double strand breaks

**FIGURE 1:**
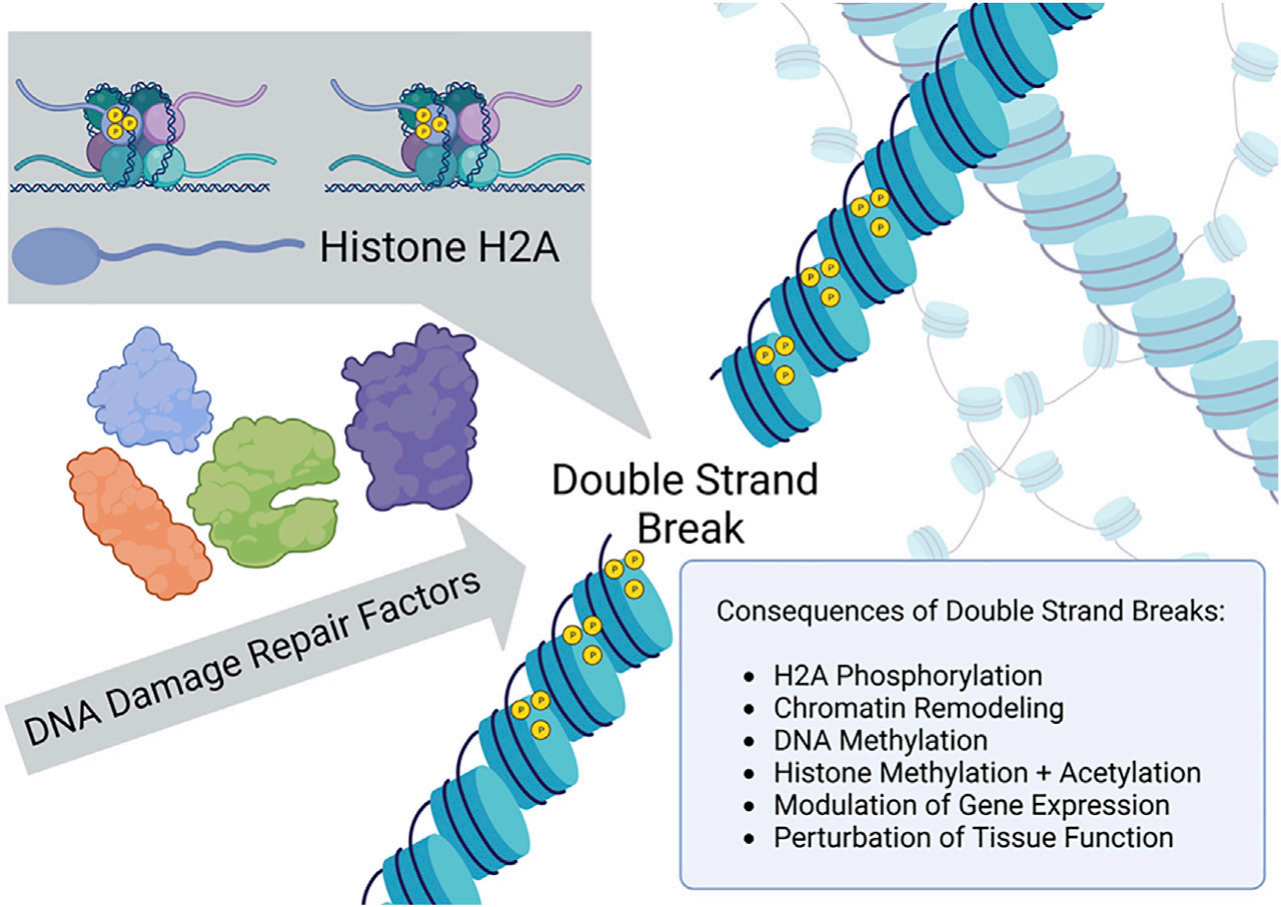
γ-H2AX recruitment to the site of a double strand break and chromatin remodeling signals epigenetic changes as a DNA damage response.

Earlier observations by Shroff et al. (2004) provided evidence that a site-specific double-strand break (DSB) introduced by an HO endonuclease ectopically expressed in yeast resulted in phosphorylation of histone 2A isoform H2AX (designated γ-H2AX) in the vicinity of the DSB and rapid recruitment of DNA repair proteins to the break site. Thus, in a conserved model eukaryotic system, chromatin phosphorylation signaling elicited by a defined DSB caused the timely re-localization of cellular DNA damage response factors to the site of the chromosomal break. A similar signaling pathway in human cells leading to activation of the DNA damage signaling kinase ATM was elucidated (Berkovich et al., 2007), suggesting that structural modifications to chromatin facilitate the recruitment of DNA repair factors necessary for efficient rejoining of the DSB. A similar system was exploited by Yang et al. (2023) to investigate the relationship of DSBs to epigenetic modifications and aging in mice (Figure 1). Although not the focus of the study by Yang et al. (2023), there has been much interest in how chromatin remodeling induced by DSBs might influence pathway choice for repair by homologous recombination or nonhomologous end-joining (Abate and Hendzel, 2022). Yang et al. more centrally examined the interplay of chromatin dynamics with transcriptional regulation, a topic of considerable relevance (Min et al., 2022), and how this influences aging.

To induce site-specific chromosomal DSBs in mammalian cells, Yang et al. (2023) employed ectopically expressed I-Pol endonuclease, an enzyme recognizing 19 non-coding regions and one ribosomal region of genomic double-stranded DNA, to create the targeted breaks without also introducing insertions or deletions. The DSBs induced γ-H2AX chromatin modifications which facilitated recruitment of DNA repair factors and resealing of the broken genomic DNA. In addition, when the chromatin structure was altered and proteins recruited to the genomic DNA proximal to the break, the epigenetic profile was modified at both the DNA and histone levels. Yang et al. verified that changes to the epigenetic landscape did indeed occur at regions surrounding the DSBs; furthermore, they investigated the hypothesis that aging phenotypes resulted from these epigenetic changes (next section). Although molecular aspects of γ-H2AX amplification signaling were not a focus of the research study by Yang et al., this phenomenon should be focused on in future work to further define mechanistic aspects of the observed *in vitro* and *in vivo* aging.

## Epigenetics and its role in aging

**FIGURE 2:**
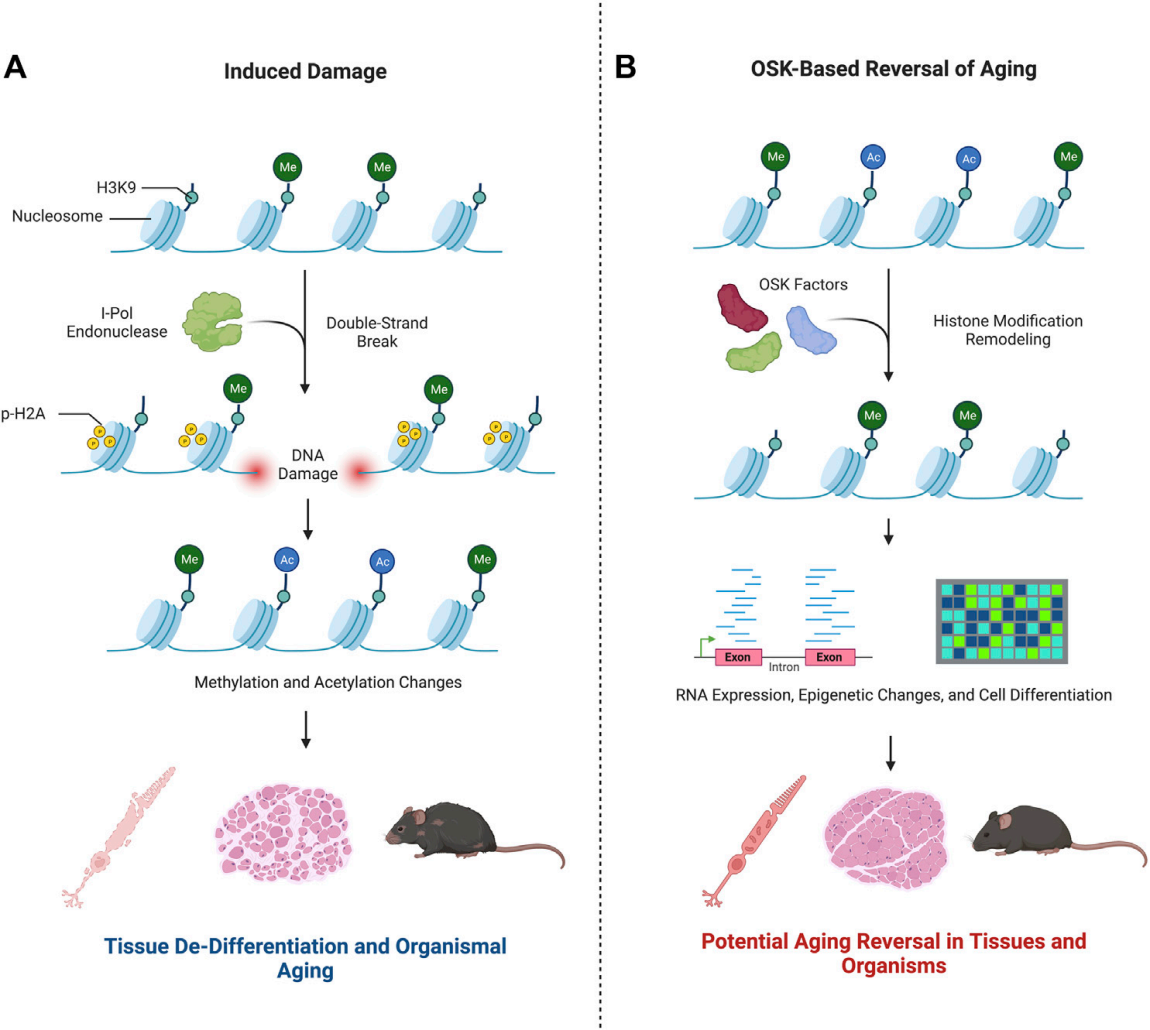
Epigenetic changes play a key role in aging. Research by Yang et al. (2023) advances the hypothesis that *in vitro* and *in vivo* aging can be modulated by changes to the epigenetic state. This is summarized in the graphic. **(A)** Double strand breaks induced by I-Pol endonuclease modify the epigenetic landscape–see text for more details. As a result, Yang et al. demonstrated a de-differentiation and aging of nervous system cells, muscles, and the mouse organism as a whole. **(B)** Upon exposure to OSK factors, the re-differentiation of the cells treated were shown to have youth-like RNAseq and epigenetic profiles. The cell-based phenotype healing of nerve and muscle cells or the whole organism were not evidenced.

Yang et al. posed the question: how can the consequences of epigenetic changes be untangled from changes due to cellular DNA damage, or *vice versa*? To address this, they devised a mechanism to induce epigenetic changes, specifically changes to 1) methylation patterns at specific CpG islands, cystine guanine dinucleotides occurring in gene promoters that are commonly methylated, and 2) acetylation and methylation of histones H3 and H4 (Hughes et al., 2020; Yang et al., 2023). This strategy perturbed two different epigenetic parameters–DNA methylation and histone modifications–in the same experimental model. Historically, it has been understood that changes in histone modifications alter how tightly DNA is wrapped around histone complexes, modifying accessibility of the genome to its own enhancers, transcription factors, and RNA polymerase (Zhang et al., 2021). In the current work, Yang et al. examined the loss of epigenetic differentiation over time as *in vitro* or *in vivo* aging (Figure 2). This idea was first introduced in a theoretical application of *in vivo* epigenetic aging changes in “The Strategy of the Genes: a Discussion of Some Aspects of Theoretical Biology” (Waddington, 1957), where Waddington presented the idea that cells are highly differentiated in their youth. Later in life, epigenetic changes cause the cells to express genes less specific to the original tissue or cell type, de-differentiating and causing them to lose their innate functions. This idea then leads to aging and eventual death. Subscribing to this theory, in their work Sinclair’s team and their collaborators accelerated this process of epigenetic change and then attempted to reverse it (Yang et al., 2023).

The approach employed by Yang et al. to induce gene expression changes characteristic of a youthful signature was achieved by ectopic expression of the transcription regulatory factors Oct4, Sox2, and Klf4 (OSK), previously shown to induce the formation of pluripotent stem cells from somatic cells (Takahashi and Yamanaka, 2006). Remarkably, Yang et al. demonstrated that ectopic expression of the OSK factors in retinal ganglion cells in 12-month-old mice restored mRNA levels to that typical of younger mice, attesting to the power of the approach to modulate gene expression *in vivo*. It is difficult to distinguish, however, if the OSK factors successfully reprogram all cells or if the reprogramming favors undamaged cells and expands those populations. If the OSK factors reprogramed cells that accumulated mutations, negative phenotypes could persist.

## Epigenetic manipulation to accelerate aging in vitro and in vivo

The findings by Yang et al. (2023) that induction of epigenetic changes accelerates *in vitro* and *in vivo* aging are impressive. The results were not exclusively associated with transcriptional or epigenetic changes, but also macroscopic organismal performance in behavioral tests, both cognitive and activity-based. Moreover, both neurological degeneration and muscular atrophy associated with natural and accelerated aging were detected (Yang et al., 2023). *In vitro*, ectopic expression of OSK factors caused reversal of gene expression changes in neural cells via alteration of histone and DNA methylation patterns to those associated with cells of a younger age (Yang et al., 2023). The reversal of epigenetic features and gene expression to a more youthful pattern brings strategies to induce pluripotency to the forefront of emerging therapies to restore a healthy aging phenotype.

## Key advances and limitations of the Yang et al. Study

The reversal of age-associated epigenetic and transcription patterns is a major advance. The researchers were able to successfully show a reversal of the RNA expression patterns using RNAseq as well as the restoration of H3K9me3 and H3K36me2 to levels associated with young cells in muscle and kidneys, though re-establishment at specific loci for chromatin modifications was not examined. However, they were able to visualize improvement in neural cells of the optic nerve, where they injected plasmid encoding OSK factors. This was also associated with changes in the RNAseq data 4 weeks later, with cells expressing a more youthful profile. Tying back to the idea of Waddington Valleys, the ICE cells, when attempting to be re-differentiated after induced aging, showed significant cell-type re-differentiation compared to the WT cells, which is considered falling back into a Waddington Valley–something that typically degrades with age.

Despite the advances, there are some limitations to the study. While the reversal of the DNA methylation age was seen in the neural cells as well as in the RNA sequence expression, no phenotypic changes were shown beyond this. Although the study delved extensively into the validation of the experimental mouse model to demonstrate accelerated aging, including cognitive and activity-based tests as well as immunohistochemistry of involved tissues, these parameters were not assessed for aging reversal. It would have been enlightening if the authors assessed the attempted reversal of aging with the OSK factors, as it pertains to life expectancy, cognitive or muscular changes, or if there were any other tissue-specific or cell-specific alterations beyond what was already documented in the ICE mouse model. While the study effectively showed the reversal of the RNAseq and DNA methylation changes in neural cells, there were no other cell types documented and it was not reported if the OSK factors re-differentiated cells in a tissue-specific manner or if they applied the same epigenetic changes to all cells after ectopic expression of the rejuvenation factors. For example, if old liver or kidney cells were exposed to the OSK factors, would they undergo the same epigenetic changes, or would the OSK factors be able to do this in a tissue-specific manner? Further, it will be of great interest in future studies to characterize the altered histone landscape more robustly at specific loci in different tissues after exposure to OSK factors to determine the extent that the epigenetic landscape is restored.

Although Yang et al. were able to demonstrate with their restriction endonuclease-inducible system that DSBs with limited single-stranded overhangs led to epigenetic changes associated with aging, it will be intriguing to determine if other forms of DNA damage such as single-strand breaks, nicks, chromosome rearrangements or oxidatively damaged bases contribute to aging via epigenetic modifications, and if these are similar to or distinct from those in the experimental system employed by Yang et al. Defining the epigenetic programming pathways altered by endogenous *versus* exogenously induced DNA damage may lead to new insights for normal aging not seen using experimental models that employ well defined and relatively clean DSBs.

## Healthspan perspective

Healthspan is of great interest to many groups engaged in aging research. As opposed to lifespan, healthspan can be defined as the period of a person’s life when they are considered to be in good health (Kaeberlein, 2018). Relating this idea of healthspan to the Yang et al. (2023) study, it would be informative to determine if the epigenetic reversal of aging increases healthspan, or simply de-differentiates a given cell type into an epigenetically younger version. This outcome may be important for human health. From a clinical perspective, a very small percentage of deaths in the United States population or around the globe are attributed to non-accidental or non-disease related (Centers, 2023). In fact, of the top ten causes for death in the United States in 2019, nine of the ten were disease-related (Centers, 2023). In essence, humans are not living to their maximum genetically capable lifespan because they succumb to disease first. As such, extending healthspan should be a welcome strategy to deter human disease.

This concept takes origin from the field of geroscience which has the aim to characterize the genetic, molecular, and cellular mechanisms underlying aging as the major risk factor and driving force of many chronic conditions and diseases manifest in individuals advanced in their years. Thus, a focus on healthy aging represents the best way to avoid onset of debilitation and disease. Arguing along these lines, it would be highly beneficial to determine if the reversal of epigenetic aging via re-differentiation of cells protects the involved tissues and organs against disease-related processes. Alternatively, if it was merely a mechanism to increase the lifespan and not healthspan, the generalizable benefit may be less.

## Conclusion

The paper by Sinclair’s lab and collaborators marks an interesting and important step towards the reversal of aging, which promises many benefits including extended lifespan. Significantly, the authors present experimental evidence in a model system to support the idea that epigenetic remodeling may be exploited to restore youthful potential. Some of the remaining questions to be answered to support this theory include evidence for 1) tissue-specific epigenetic reversal, 2) cognitive and muscular aging reversal via the same tests used in the initial experimental validation, 3) alterations to lifespan, and 4) resistance to disease or other hazards in the mouse model. Collectively, addressing these parameters would further support the idea of extending lifespan by epigenetic manipulations.

The study by Yang et al. was performed exclusively with mouse models, and it will be exciting to see if these findings would apply to other model organisms or humans. Importantly, an assessment of age-related clinical features and their reversibility by an epigenetic mechanism would provide new hope for research in the treatment of age-related diseases.

## Data Availability

The original contributions presented in the study are included in the article/Supplementary Material, further inquiries can be directed to the corresponding author.
